# Maternal intervention with a combination of galacto-oligosaccharides and hyocholic acids during late gestation and lactation increased the reproductive performance, colostrum composition, antioxidant and altered intestinal microflora in sows

**DOI:** 10.3389/fmicb.2024.1367877

**Published:** 2024-06-12

**Authors:** Jian Yu, Jie Wang, Chang Cao, Jiani Gong, Jiaqi Cao, Jie Yin, Shusong Wu, Peng Huang, Bi’e Tan, Zhiyong Fan

**Affiliations:** College of Animal Science and Technology, Hunan Agricultural University, Changsha, China

**Keywords:** sow, galacto-oligosaccharides, hyocholic acids, reproductive performance, gut microbiota

## Abstract

**Introduction:**

This study was conducted to evaluate the effects of dietary galacto-oligosaccharides (GOS) and hyocholic acids (HCA) during late gestation and lactation on reproductive performance, colostrum quality, antioxidant capacity and gut microbiota in multiparous sows.

**Methods:**

A total of 60 healthy multiparous cross-bred sows (Landrace × Yorkshire) were randomly fed 4 groups diets as follows: the basal diets (CTRL group), or the basal diets containing only 600 mg/kg GOS (GOS group), 600 mg/kg GOS + 100 mg/kg HCA (GOS + Low HCA group), and 600 mg/kg + 200 mg/kg HCA (GOS + High HCA group) from d 85 of gestation to weaning. Multiple parameters of sows were determined.

**Results:**

There was a trend of shortening the labor process of sows (*p* = 0.07) in the GOS group and GOS + Low/High HCA group. Compared with the CTRL group, the GOS + Low/High HCA group increased the average piglets weight at birth (*p* < 0.05), and increased the IgA concentration of colostrum (*p* < 0.05). In addition, serum triglyceride (TG) concentration was lower (*p* < 0.05), and serum total antioxidant capacity (T-AOC) was higher (*p* < 0.05) in the GOS and GOS + Low/High HCA groups than in the CTRL group at farrowing. Serum catalase (CAT) activities was higher in the GOS and GOS + High HCA groups than in the CTRL group at farrowing. The 16S rRNA analysis showed that GOS combination with high-dose HCA shaped the composition of gut microbiota in different reproductive stages (d 107 of gestation, G107; d 0 of lactation, L0; d 7 of lactation, L7). At the phylum level, the relative abundance of *Bacteroidota* and *Desulfobacterota* in G107, *Bacteroidota*, and *Proteobacteria* in L0, and *Planctomycetota* in L7 was increased in GOS + High HCA group (*p* < 0.05). Spearman correlation analysis showed that *Streptococcus* was positively correlated with the serum TG but negatively correlated with the average piglets weight at birth (*p* < 0.05).

**Conclusion:**

This investigation demonstrated that the administration of galacto-oligosaccharides (GOS) in conjunction with hyocholic acids (HCA), to sows with nutrient restrictions during late gestation and lactation, further improved their antioxidant capacity and milk quality. The observed beneficial effects of GOS + HCA supplementation could potentially be linked to an improvement in gut microbiota disorders of the sows.

## 1 Introduction

The pivotal role of breeding sows in the pig industry is well known, particularly in terms of their health and reproductive performance. However, sows are vulnerable to major immune system and physiological metabolism changes during pregnancy, including heightened oxidative stress and inflammatory response, which can result in blood levels of pro-inflammatory factors like interleukin-6, tumor necrosis factor, and reactive oxygen species being elevated (Berchieri et al., 2011). Constipation, miscarriages, and intrauterine development retardation are among the reproductive disorders that are intimately linked to an imbalance in the inflammatory response. Therefore, in order to improve sows’ reproductive success, it is crucial to boost their immunity throughout the late gestation phase.

As a natural functional oligosaccharide, galacto-oligosaccharides (GOS) are not easily digested and hydrolyzed but are fermented by microflora in hindgut to produce short-chain fatty acids (Vos et al., 2007). Furthermore, supplementation with GOS has been indicated to stimulate the proliferation and/or activity of beneficial bacteria, especially *Bifidobacteria* and *Lactobacillus* (Gopal et al., 2001; Monteagudo et al., 2016). In fact, research data have demonstrated that dietary supplementation of GOS in pregnancy sows can elevate the plasma immunoglobulin A to enhance the immune status (Wu et al., 2021; Lee et al., 2023).

It has recently been established that bile acids (BAs), which are amphipathic chemicals produced by the liver’s catabolism of cholesterol, are important signaling molecules in the body. They play a role in several physiological processes, such as immunological regulation, hepatic insulin resistance, energy balance, lipid and glucose metabolism, and bile acid metabolism (Zong et al., 2019; Cao et al., 2021). This recognition primarily stems from their ability to activate specific bile acids receptors, as evidenced in recent studies (Molinaro et al., 2018; Ahmad and Haeusler, 2019). A great deal of study has been done on feeding BAs to weaned pigs. These investigations have shown that taking such supplements might enhance humoral immune responses and raise blood antioxidant capacity.

However, there is a paucity of research exploring the potential benefits of dietary mixtures of GOS and bile acids (BAs) on the health of suckling piglets via maternal nutrition. Given the complementary and synergistic biological functions of GOS and BAs, it is hypothesized that incorporating a combination of these substances into the diets of perinatal sows could markedly enhance reproductive performance, bolster antioxidant capacity, and improve colostrum quality through modulation of the gut microbiota.

## 2 Materials and methods

### 2.1 Ethics approval

All animal care procedures in our study were approved by the Institution of Animal Care and Use Committee of the College of Animal Science and Technology, at Hunan Agricultural University (Changsha, China), and were conducted in accordance with the National Institutes of Health (Changsha, China) guidelines for the care and use of experimental animals.

### 2.2 Galacto-oligosaccharides (GOS) and bile acids (BAs)

The GOS (product name: Kang Liwei, purity ≥88%, moisture ≤2%, light yellow, fine sand) was provided by the Chengdu Associated Bio-Technology Co., Ltd. (Chengdu, China). The BAs were hyocholic acids (product name: glycinocholic acid sodium salt, total acidity ≥85%, moisture ≤2%, white, powder) and were provided by the Wuhan Huaxiang Kejie Bio-Technology Co., Ltd. (Wuhan, China).

### 2.3 Animals and experimental design

Third to fifth parity late pregnancy sow (*n* = 60; d 85 of gestation, Landrace × Yorkshire) with similar backfat thickness and an average body weight of 302 ± 13 kg were randomly assigned to one of 4 treatments: the basal diets (diets without GOS and HCA, *n* = 15, CTRL group), basal diets containing only 600 mg/kg GOS (*n* = 15, GOS group), basal diets containing 600 mg/kg GOS + 100 mg/kg HCA (*n* = 15, GOS+ Low HCA group), and basal diets containing 600 mg/kg GOS + 200 mg/kg HCA (*n* = 15, GOS + High HCA group). The basal diets (Table 1) were divided into a pregnancy and a lactation diet and nutritional requirements were formulated on the basis of the recommendations by the National Research Council (National Research Council, 2012). Sows were raised individually in stalls (2.0 m × 0.6 m) with concrete-floored from d 85 to 107 and fed a total of 3 kg diet daily (8:30 and 15:30). Then, the sows were shifted to farrowing stalls (2.13 m × 0.66 m) with concrete-floored and offered 2 kg of daily diet (8:30 and 15:30) during d 108 of gestation to delivery. After farrowing, all sows were switched to lactation diets and had free access to the diets until weaning. In the whole experiment procedure, sows and piglets were allowed to drink water freely. During the entire experiment, laboratory animals and pigsty were provided by Chenfeng farm of Hunan Yangxiang Agriculture and Animal Husbandry Co., Ltd., and feeding management and immunization procedures of sows and piglets were carried out according to the company’s standards.

**Table 1:** Composition and nutrient contents of basal diets (as-fed basis, %).

Ingredients	Content	
	Late gestation	Lactation
Corn	5.40	20.00
Red Sorghum	15.00	10.00
Wheat	–	29.20
Hulled barley	54.60	13.20
Wheat bran	15.00	–
Soybean meal (43% crude protein)	5.55	20.30
Soybean oil		2.50
L-Lys-HCI (98%)	0.28	0.38
Limestone	1.81	1.70
Dicalcium phosphate	0.65	1.2
Salt	0.40	0.40
Vitamin-mineral premix^a^	1.31	1.12
Total	100	100
Nutrient composition^b^		
Digestible energy, kcal/kg	3,022	3,471
Crude protein, %	12.06	17.15
Crude fiber, %	3.18	2.63
Calcium, %	0.85	0.76
Total phosphorus, %	0.55	0.61
Available phosphorus, %	0.33	0.43
Total lys, %	0.79	1.26

a The premix provided the following per kilogram of complete diets: Zn, 80.0 mg; Cu, 6.0 mg; Fe, 80.0 mg; Mn, 20.0 mg; I, 0.15 mg; Se, 0.15 mg; vitamin A, 10,000 IU; vitamin D_3_, 2,100 IU; vitamin E, 45 IU; vitamin K_3_, 4 mg; vitamin B_1_, 2.0 mg; vitamin B_2_, 8.0 mg; vitamin B_6_, 4.0 mg; vitamin B_12_, 0.025 mg; nicotinic acid, 25.0 mg; pantothenic acid, 20.0 mg; folic acid, 1.0 mg; biotin 0.5 mg.

b The nutrient levels of crude protein, crude fiber, calcium and total phosphorus were measured values; other values were calculated.

### 2.4 Sample collection and processing

Basal diets were ground to pass through a 0.5 mm screen using a mill grinder. Crude protein, crude fiber, calcium and total phosphorus contents of the diets were determined according to AOAC (2007).

Fresh feces of the sows (*n* = 6 per group) were collected individually by massaging the rectum at d 107 of gestation (d 7 before delivery), and d 0 and d 7 of lactation (before each sampling, the buttocks of the sows were washed with 1/1000 potassium permanganate, the anus was disinfected with alcohol cotton balls). Next, 24 fecal samples were transported to the laboratory and stored at −80°C until later analysis.

Six sows per group were randomly selected for blood samples. A 10 mL blood was collected into centrifuge tubes from the sows’ ear veins on the farrowing day (within 2 h of delivery). The serum samples were obtained by centrifuging blood samples at 3000 r/min for 15 min. Whereafter they were stored at −20°C for the analysis.

On the farrowing day (within 2 h of delivery), the mixed colostrum samples (*n* = 6) were collected from the anterior, middle and posterior three milk areas of the sows. Approximately 10 mL of colostrum per sow was collected into a centrifuge tube. The samples were rapidly stored at −20°C for the analysis.

### 2.5 Determination of redox status of serum

Total antioxidant capability (T-AOC), the activities of superoxide dismutase (SOD), catalase (CAT), total glutathione (T-GSH) and malonaldehyde (MDA) in serum were estimated using commercial kits (Nanjing Jiancheng Bioengineering Institute, Nanjing, China), according to the manufacturer’s protocols with a V1600 Split Beam Visible Spectrophotometer (Meipuda Co., Shanghai, China). The results were expressed as units per milliliter of serum.

### 2.6 Determination of milk composition and immunoglobulin

Colostrum samples were analyzed for total cholesterol (TC) and triglyceride (TG) using a fully automated biochemical analyzer. Whey samples were obtained by centrifuging colostrum samples at 4000 r/min for 10 min, and the concentration of immunoglobulin A (IgA) was analyzed by using commercial kits (Cusabio Biotech Co., Ltd., Wuhan, China), according to the manufacturer’s protocols.

### 2.7 Determination of serum metabolites

Glucose (GLU), triglyceride (TG), total cholesterol (TC), high-density lipoprotein cholesterol (HDL-C) and low-density lipoprotein cholesterol (LDL-C) were determined using reagent kits (Nanjing Jiancheng Bioengineering Institute, Nanjing, China). All of the procedures were carried out in accordance with the manufacturer’s protocols.

### 2.8 DNA extraction, PCR amplification, and bacterial 16S ribosomal RNA (rRNA) gene sequencing

The total genomic DNA was extracted from fecal samples of sows (at d 107 of gestation, at farrowing and at d 7 of lactation) using a DNA kit (LC-Bio Technology Co., Ltd., Hangzhou, China). The quality of isolated DNA was determined by agarose gel electrophoresis. Subsequently, the V3-V4 hypervariable region of the bacterial 16S rRNA gene was used as a template for PCR amplification. The V3-V4 gene region of 16S rRNA was amplified by using the forward primer 338F: 5’-ACTCCTACGGGAGGCAGCAG-3′ and 806R: 5’-GGACTACHVGGGTWTCTAAT-3′. For each sample, a 8-digit barcode sequence was added to the 5′ end of the forward and reverse primers (provided by Allwegene Company, Beijing). The volume of the PCR was 25 μL and included 12.5 μL 2 × Taq PCR MasterMix (Vazyme Biotech Co., Ltd., China), 3 μL BSA (2 ng/μL), 1 μL Forward Primer (5 μM), 1 μL Reverse Primer (5 μM), 2 μL template DNA, and 5.5 μL double distilled H_2_O. Cycling parameters were 95°C for 5 min, followed by 28 cycles of 95°C for 45 s, 55°C for 50 s and 72°C for 45 s with a final extension at 72°C for 10 min. The PCR products were purified using a Agencourt AMPure XP Kit (Beckman Coulter, Inc., United States). Sequencing libraries were generated using NEB Next Ultra II DNA Library Prep Kit (New England Biolabs, Inc., United States) following the manufacturer’s recommendations. The sequences were clustered into operational taxonomic units (OTU) at a similarity level of 97% to generate rarefaction curves and to calculate the richness and diversity indices.

### 2.9 Statistical analysis

The sows were treated as an experimental unit in all of the statistical analyses. First tests for normal distribution and homogeneity of variance were performed on the data. Subsequently, the data between different groups were analyzed by a one-way ANOVA and Duncan’s multiple range test using SPSS 26.0 software (SPSS Inc., Chicago, IL, United States). GraphPad Prism 8 (San Diego, CA, United States) was used to plot Figures. The correlation between gut microbiota and the detection indexes of sows was analyzed with R software (version. 3. 5. 1). Results were shown as means ± standard error. Probability values <0.05 and < 0.01 were considered statistically significant and highly significant, respectively.

## 3 Results

### 3.1 Effect of GOS and HCA supplementation on reproductive performance of the sows

The results of reproductive performance are shown in Table 2. There were no differences (*p* > 0.05) in Total pigs born, pigs born alive, pigs born robust, stillbirth number, mummy number, and coefficient variation of piglet litter among the four groups. Similarly, treatment differences in pig number at weaned, litter weight at weaned, and coefficient of variation at weaned were small and not important (*p* > 0.10). However, compared with the basal diets, there was a trend for the labor process from sows fed GOS and GOS + Low/High HCA diets to be shorter (0.05 < *p* < 0.10). Sows fed the GOS + Low/High HCA diets increased the average piglets weight at birth compared with sows fed the basal diets (*p* < 0.05).

**Table 2:** Effects of galacto-oligosaccharides (GOS) and hyocholic acids (HCA) on the reproductive performance of sows.

Items	Maternal treatment^1^				*p*-value
	CTRL	GOS	GOS + Low HCA	GOS + High HCA	
Parturition					
Labor process, h	3.80 ± 0.31	2.48 ± 0.64	1.62 ± 0.47	2.34 ± 1.27	0.07
Number of piglets born per litter	14.93 ± 1.00	16.20 ± 1.27	14.00 ± 0.86	15.47 ± 0.94	0.50
Number of piglets born alive per litter	13.31 ± 0.90	15.40 ± 1.05	13.08 ± 0.38	14.73 ± 0.86	0.35
Number of piglets born robust per litter	12.92 ± 0.90	15.27 ± 1.02	12.85 ± 0.39	14.27 ± 0.85	0.11
Number of stillbirths per litter	0.53 ± 0.17	0.47 ± 0.19	0.36 ± 0.17	0.50 ± 0.17	0.90
Average piglets weight at birth, kg	1.13 ± 0.10^b^	1.34 ± 1.17^ab^	1.45 ± 0.06^a^	1.49 ± 0.06^a^	0.02
Intralitter coefficient of variation of piglets at birth, %	0.21 ± 0.01	0.15 ± 0.02	0.17 ± 0.01	0.16 ± 0.01	0.24
Weaning (21 d of age)					
Number of piglets weaned per litter	11.18 ± 0.54	12.14 ± 0.57	12.02 ± 0.26	12.00 ± 0.40	0.29
Average piglets weight at weaning, kg	7.32 ± 0.15	7.40 ± 0.18	7.31 ± 0.22	7.57 ± 0.24	0.11
Average litter weight at weaning, kg	81.97 ± 3.75	89.85 ± 4.48	88.06 ± 4.41	90.74 ± 4.20	0.64
Average litter weight gain, kg	65.36 ± 3.16	69.40 ± 3.97	69.43 ± 3.55	70.05 ± 4.45	0.47
Intralitter coefficient of variation of piglets at weaning, %	0.13 ± 0.02	0.12 ± 0.01	0.10 ± 0.01	0.13 ± 0.01	0.43

^1^ CTRL = basal diets; GOS = basal diets +600 mg/kg GOS; GOS + Low HCA = basal diets +600 mg/kg GOS + 100 mg/kg HCA; GOS + High HCA = basal diets +600 mg/kg GOS + 200 mg/kg HCA. Values are expressed as means ± standard error, *n* = 15 for each group. ^a, b^ Mean values within a row with different letter superscripts are significantly different (*p* < 0.05).

### 3.2 Effect of GOS and HCA supplementation on concentrations of serum and colostrum metabolites

As reported in Table 3, GOS and GOS + Low/High HCA supplementation decreased the serum TG concentration of sows at delivery (*p* < 0.05), and the concentration of GLU increased slightly (0.05 < *p* < 0.10). However, the sows were not observed distinct differences in serum LDL-C, HDL-C, TC and TBA concentrations between the CTRL group and GOS or GOS + Low/High HCA groups at delivery (*p* > 0.10).

**Table 3:** Effect of galacto-oligosaccharides (GOS) and hyocholic acids (HCA) on serum metabolites levels of sows at farrowing.

Items^2^	Maternal treatment^1^				*p*-value
	CTRL	GOS	GOS + Low HCA	GOS + High HCA	
LDL-C, mmol/L	0.42 ± 0.02	0.48 ± 0.04	0.46 ± 0.04	0.46 ± 0.04	0.83
HDL-C, mmol/L	0.66 ± 0.03	0.60 ± 0.04	0.58 ± 0.04	0.62 ± 0.03	0.34
TC, mmol/L	1.61 ± 0.08	1.56 ± 0.08	1.52 ± 0.06	1.57 ± 0.08	0.85
TG, mmol/L	0.55 ± 0.02^a^	0.30 ± 0.02^b^	0.31 ± 0.03^b^	0.34 ± 0.05^b^	<0.01
TBA, mmol/L	74.37 ± 3.59	56.77 ± 11.22	54.83 ± 4.55	59.99 ± 12.84	0.38
GLU, mmol/L	4.02 ± 0.15	4.58 ± 0.55	5.18 ± 0.22	5.24 ± 0.35	0.08

^1^ CTRL = basal diets; GOS = basal diets +600 mg/kg GOS; GOS + Low HCA = basal diets +600 mg/kg GOS + 100 mg/kg HCA; GOS + High HCA = basal diets +600 mg/kg GOS + 200 mg/kg HCA. Values are expressed as means ± standard error, *n* = 15 for each group. ^2^ LDL-C = low density lipoprotein cholesterol; HDL-C = high density lipoprotein cholesterol; TC = total cholesterol; TG = triglyceride; TBA = total bile acids; GLU = glucose. ^a, b^ Mean values within a row with different letter superscripts are significantly different (*p* < 0.05).

As shown in Table 4, it was found that the concentrations of TG on colostrum increased slightly in the GOS and GOS + Low/High HCA supplementation groups compared with the CTRL group at farrowing (0.05 < *p* < 0.10). In addition, the IgA concentration of colostrum was significantly increased by dietary GOS + Low/High HCA supplementation (*p* < 0.05).

**Table 4:** Effect of galacto-oligosaccharides (GOS) and hyocholic acids (HCA) on colostrum metabolites and immunoglobulin a levels of sows at farrowing.

Items^2^	Maternal treatment^1^				*p-*value
	CTRL	GOS	GOS + Low HCA	GOS + High HCA	
TC, mmol/L	3.24 ± 0.18	3.51 ± 0.26	3.76 ± 0.31	3.29 ± 0.18	0.41
TG, mmol/L	30.90 ± 3.29	36.00 ± 1.70	37.16 ± 0.95	37.17 ± 1.10	0.08
IgA, mg/mL	375878.67 ± 54654.21^b^	314293.33 ± 24431.04^b^	556164.00 ± 67100.28^a^	599001.33 ± 28031.05^a^	0.01

^1^ CTRL = basal diets; GOS = basal diets +600 mg/kg GOS; GOS + Low HCA = basal diets +600 mg/kg GOS + 100 mg/kg HCA; GOS + High HCA = basal diets +600 mg/kg GOS + 200 mg/kg HCA. Values are expressed as means ± standard error, *n* = 15 for each group. ^2^ TC = total cholesterol; TG = triglyceride; IgA = immunoglobulin A. ^a, b^ Mean values within a row with different letter superscripts are significantly different (*p* < 0.05).

### 3.3 Effect of GOS and HCA supplementation on serum antioxidant capacity of sows

The results of serum antioxidant indexes are reported in Table 5. Compared with the control sows, GOS and GOS + Low/High HCA supplementation increased serum T-AOC (*p* < 0.05). At the same time, sows in the GOS and GOS + High HCA groups had higher activities of CAT (*p* < 0.05) than sows in the CTRL group. There was no evident difference in the activities of serum MDA between the four groups (0.05 < *p* < 0.10).

**Table 5:** Effects of galacto-oligosaccharides (GOS) and hyocholic acids (HCA) on serum antioxidant index of sows at farrowing.

Items^2^	Maternal treatment^1^				*p*-value
	CTRL	GOS	GOS + Low HCA	GOS + High HCA	
T-AOC, U/mL	0.75 ± 0.02^b^	0.78 ± 0.01^a^	0.80 ± 0.01^a^	0.79 ± 0.01^a^	0.03
T-GSH, μmol/L	3.30 ± 0.85	4.26 ± 0.47	3.39 ± 0.39	5.39 ± 0.65	0.11
CAT, U/mL	1.33 ± 0.17^c^	4.87 ± 0.63^a^	2.92 ± 0.72^bc^	3.34 ± 0.46^ab^	<0.01
SOD, U/mL	22.79 ± 1.69	26.32 ± 0.84	25.85 ± 1.55	26.91 ± 1.11	0.17
MDA, nmol/mL	3.17 ± 0.57	1.90 ± 0.28	3.07 ± 0.34	2.39 ± 0.22	0.09

^1^ CTRL = basal diets; GOS = basal diets +600 mg/kg GOS; GOS + Low HCA = basal diets +600 mg/kg GOS + 100 mg/kg HCA; GOS + High HCA = basal diets +600 mg/kg GOS + 200 mg/kg HCA. Values are expressed as means ± standard error, *n* = 15 for each group. ^2^ T-AOC = total antioxidant capacity; T-GSH = total glutathione; CAT = catalase; SOD = superoxide dismutase; MDA = malondialdehyde. ^a, b^ Mean values within a row with different letter superscripts are significantly different (*p* < 0.05).

### 3.4 Effect of GOS and HCA supplementation on community composition of microbiota at phyla or genera level and alpha-diversity

**Figure 1:**
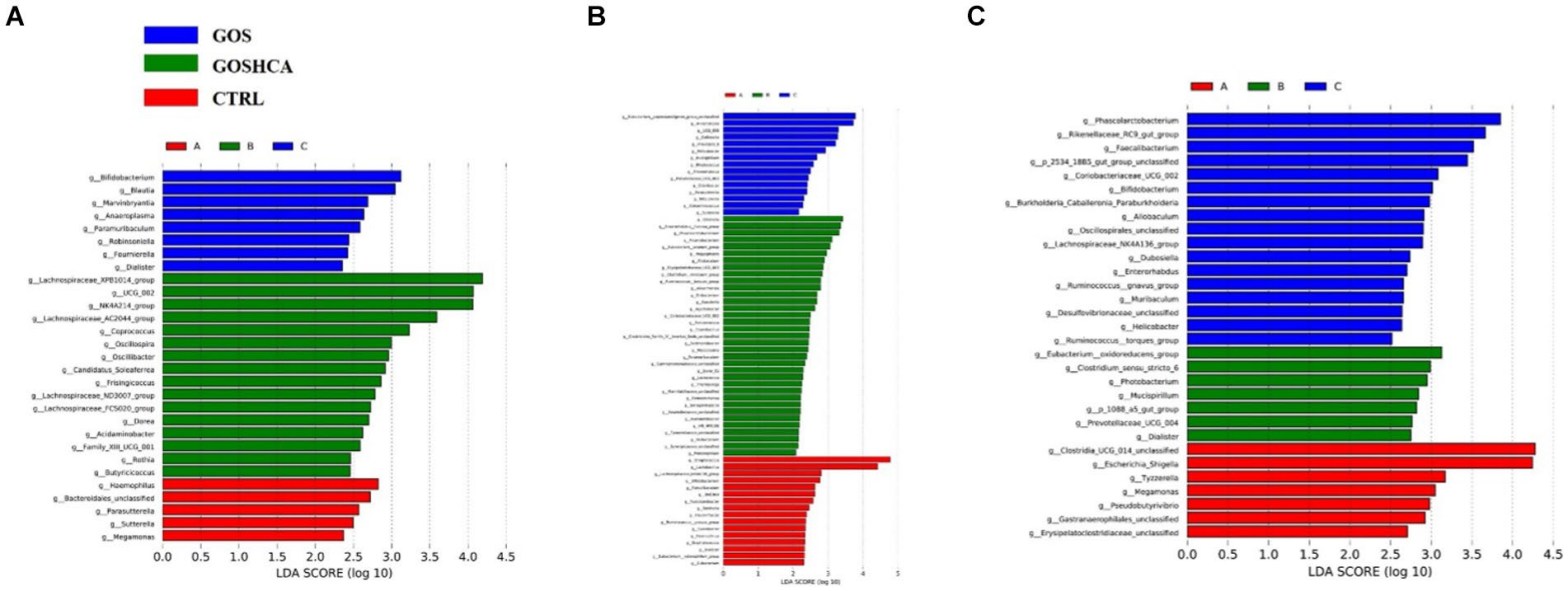
LEfSe analysis of the gut microbiota composition of sows at d 107 of gestation, at farrowing, and at d 7 of lactation. At d 107 of gestation, histogram of the Linear Discriminant Analysis (LDA) scores reveals the most differentially abundant taxa among different dietary treatment **(A)**. At farrowing, histogram of the LDA scores reveals the most differentially abundant taxa among different dietary treatment **(B)**. At d 7 of lactation, histogram of the LDA scores reveals the most differentially abundant taxa among different dietary treatment **(C)**. CTRL = basal diets; GOS = basal diets +600 mg/kg GOS; GOS + HCA = basal diets +600 mg/kg GOS + 200 mg/kg HCA.

According to the differences in reproductive performance, serum metabolite concentration, and serum antioxidant indexes of sows among the four groups, 16S rRNA sequencing was performed on fecal microflora of sows (at d 107 of gestation, at farrowing and at d 7 of lactation) in the CTRL group, GOS group, and GOS+ High HCA group to determine their diversity and composition. GOS combined with high-dose HCA changed the gut microbiota diversity of sows at d 107 of gestation, at farrowing and at d 7 of lactation, but the trend was different for these 3 time points. All 54 fecal samples were subjected to 16S rRNA gene sequencing (Supplementary Table S1). 1,534,842 raw_tages, 1,510,440 raw_tages and 1,510,290 raw_tages were filtered to obtain 1,350,659, 1,336,069 and 1,347,266 valid_tages at d 107 of gestation, at farrowing and at d 7 of lactation. On the basis of 97% sequence similarity, 870, 1,004 and 969 OTUs were obtained at d 107 of gestation, at farrowing and at d 7 of lactation, respectively. Next, variations in the microbial composition of all groups were explored. Linear discriminant analysis effect size (LEfSe) analysis of the bacterial community was used to filter the significantly different OTUs among groups, and the results demonstrate that there were dramatic differences in microbial composition between the treatment groups and the CTRL group (Figure 1).

**Figure 2:**
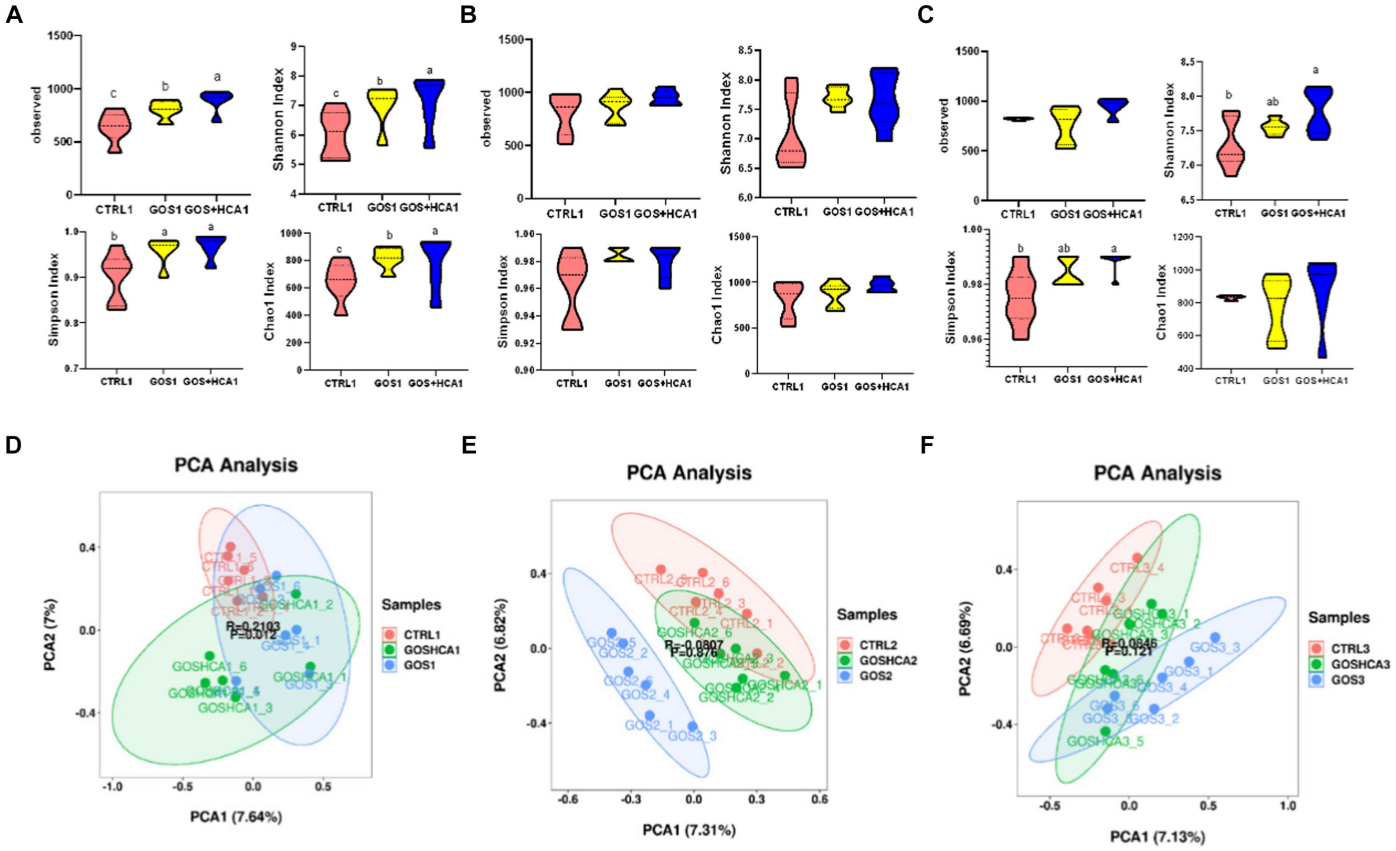
Effect of galacto-oligosaccharides (GOS) and hyocholic acids (HCA) on fecal diversity of sows. at d 107 of gestation **(A)**, at farrowing **(B)** and at d 7 of lactation **(C)**, comparison of the number of gut microbiota *α*-diversity containing bias-corrected Chao richness estimator (Chao 1), observed species, Shannon diversity indices and Simpson diversity indices among sows subjected to different dietary treatments. CTRL = basal diets; GOS = basal diets +600 mg/kg GOS; GOS + HCA = basal diets +600 mg/kg GOS + 200 mg/kg HCA. At d 107 of gestation **(D)**, at farrowing **(E)** and at d 7 of lactation **(F)**, principal component analysis (PCA) based on operational taxonomic units (OTU) among samples of different groups. Each point represents 1 sample.

In the present study, the bacterial diversity (Shannon and Simpson) and richness estimators (Chao 1 and Observed) were higher for sows in the GOS and GOS + High HCA groups (*p* < 0.05) compared with sows in the CTRL group at d 107 of gestation (Figure 2A). However, there was no difference between any of the groups regards to the Chao 1 values, number of observed species, and Shannon and Simpson indices (*p* > 0.05) at farrowing (Figure 2B). The sows fed diets containing GOS + High HCA had higher Shannon and Simpson diversity indices (*p* < 0.05) compared with the sows fed basal diets (Figure 2C). Using principal component analysis (PCA) based on OTUs, it was found that the gut microbiota of sows in the GOS and combined GOS and high-dose HCA supplementation groups were distinctly segregated from those in the CTRL group at d 107 of gestation, at farrowing and d 7 of lactation (Figures 2D–F).

**Figure 3:**
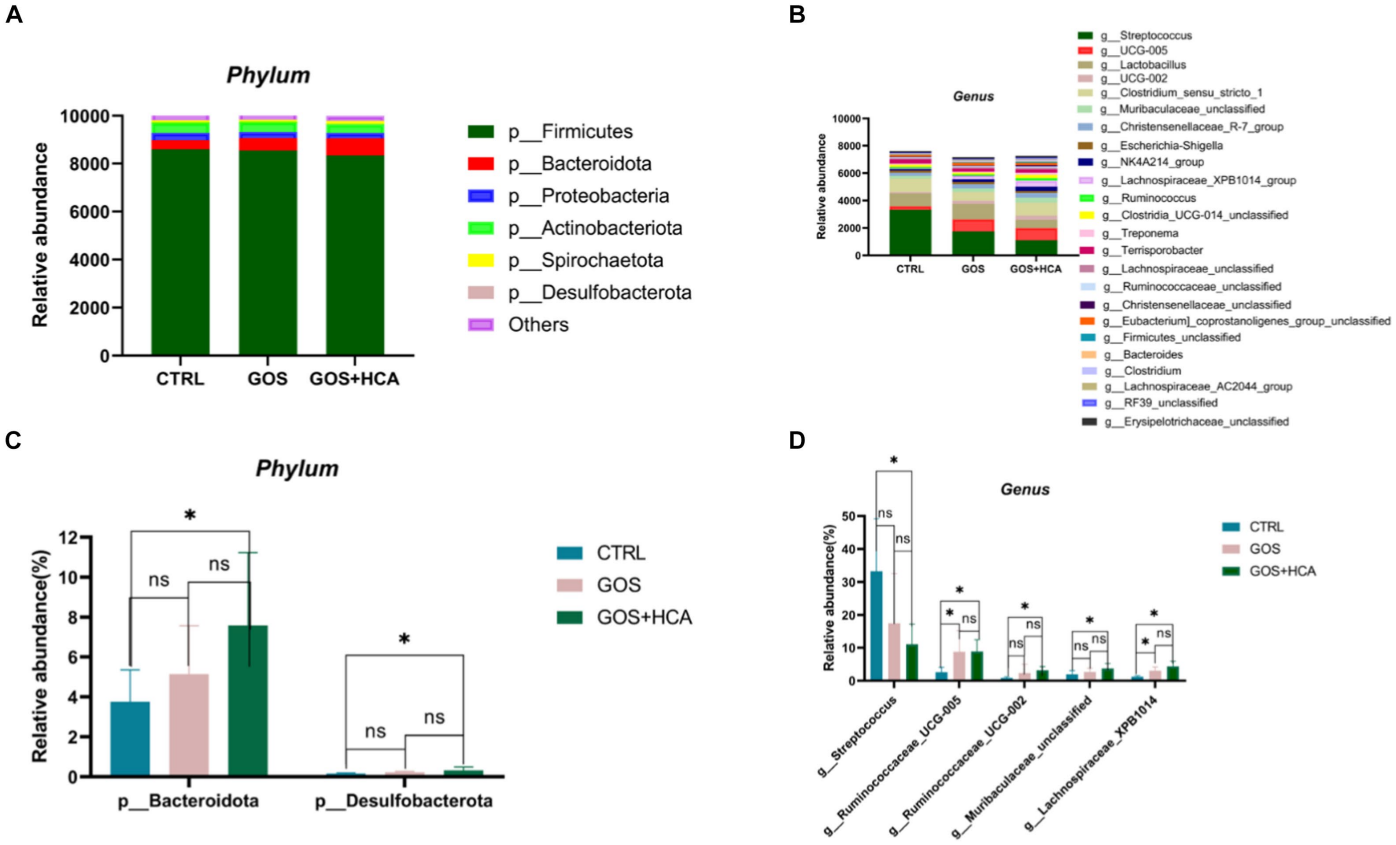
Effects of galacto-oligosaccharides (GOS) and hyocholic acids (HCA) on gut microbiota composition of sows at d 107 of gestation. Relative abundance at the phylum level of sows **(A)**, Relative abundance at the genus level of sows **(B)**. Significance test at the phylum level of sows **(C)**, significance test at the genus level of sows **(D)**. CTRL = basal diets; GOS = basal diets +600 mg/kg GOS; GOS + HCA = basal diets +600 mg/kg GOS + 200 mg/kg HCA, *n* = 6 for each group. * is significantly different (*p* < 0.05), whereas ns is not different (*p* > 0.05).

The effect of dietary supplementation of GOS and HCA on gut microbiota composition of sows at d 107 of gestation in Figure 3. At the phylum level, the abundance of *Bacteroidota* and *Desulfobacterota* was higher in the GOS+ HCA group sows, compared with sows in the CTRL group (Figure 3A). At the genus level, the abundance of *g_Ruminococcaceae_UCG005* (*p* < 0.05) and *g_Lachnospiraceae_XPB1014* (*p* < 0.05) was greater in the treatment groups than in the CTRL group (Figure 3D). Besides this, the abundance of *g_Ruminococcaceae_UCG002* and *g_Muribaculaceae_unclassified*, in the GOS + HCA group increased significantly (*p* < 0.05), but *Streptococcus* reduced significantly (*p* < 0.05).

**Figure 4:**
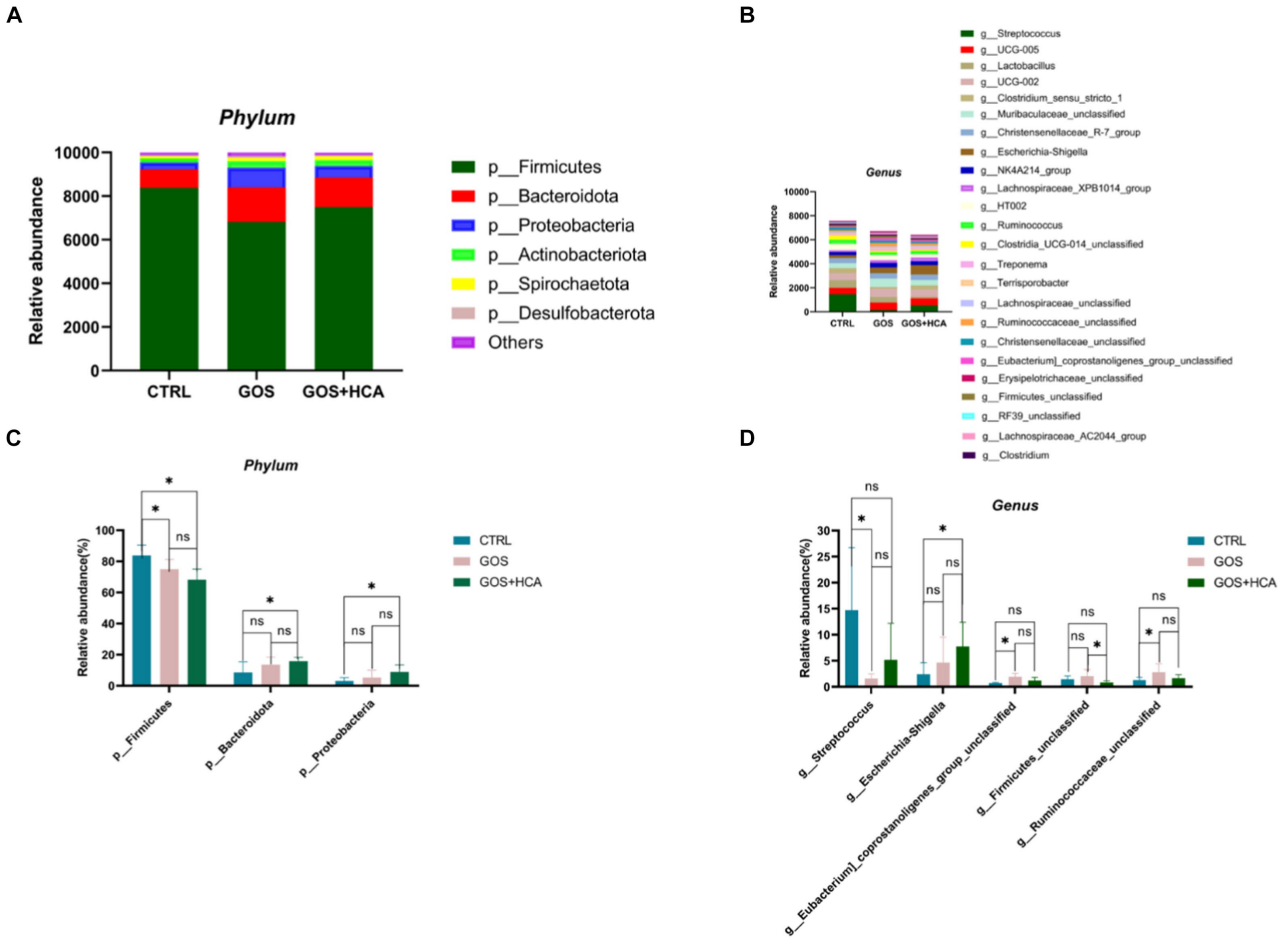
Effects of galacto-oligosaccharides (GOS) and hyocholic acids (HCA) on gut microbiota composition of sows at farrowing. Relative abundance at the phylum level of sows **(A)**, relative abundance at the genus level of sows **(B)**. Significance test at the phylum level of sows **(C)**, significance test at the genus level of sows **(D)**. CTRL = basal diets; GOS = basal diets +600 mg/kg GOS; GOS + HCA = basal diets +600 mg/kg GOS + 200 mg/kg HCA, *n* = 6 for each group. * is significantly different (*p* < 0.05), whereas ns is not different (*p* > 0.05).

The effect of dietary supplementation of GOS and HCA on gut microbiota composition of sows at farrowing in Figure 4. At the phylum level (Figure 4C), the abundances of *Bacteroidota* and *Proteobacteria* were higher (*p* < 0.05), and the abundance of *Firmicutes* was lower (*p* < 0.05) in sows in the GOS+ HCA group, compared with sows in the CTRL group at farrowing. At the genus level (Figure 4D), the abundance of *g_Escherichia-Shigella* was higher (*p* < 0.05) in sows in the GOS + HCA group compared with the CTRL group. The abundances of *g_Eubacterium_coprostanoligenes_group_unclassified* and *g_Ruminococcaceae_ unclassified* were higher (*p* < 0.05), but the abundance of *g_Streptococcus* was lower (*p* < 0.05) in sows in the GOS group, compared with the CTRL group.

**Figure 5:**
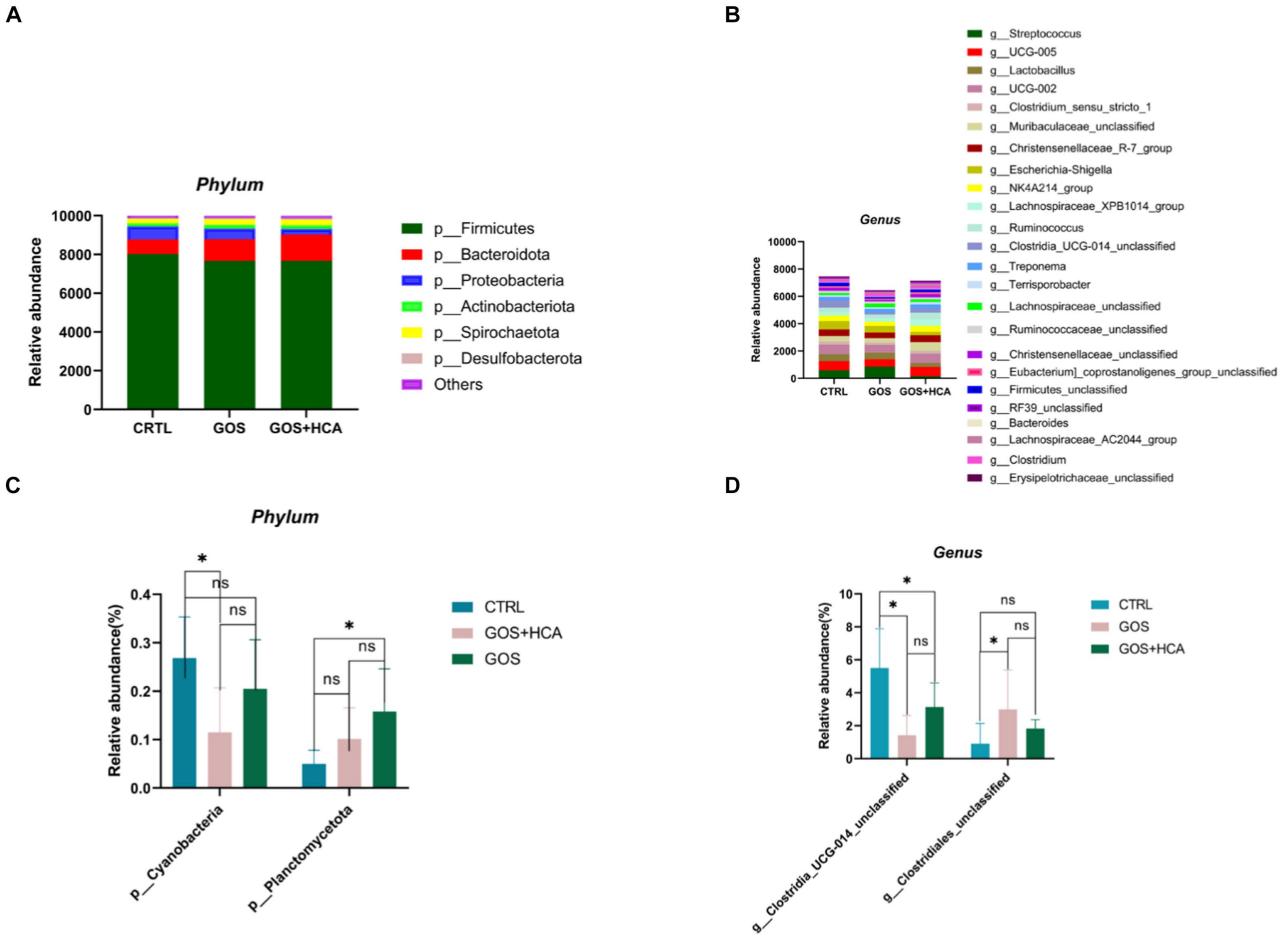
Effects of galacto-oligosaccharides (GOS) and hyocholic acids (HCA) on gut microbiota composition of sows at d 7 of lactation. Relative abundance at the phylum level of sows **(A)**, relative abundance at the genus level of sows **(B)**. Significance test at the phylum level of sows **(C)**, significance test at the genus level of sows **(D)**. CTRL = basal diets; GOS = basal diets +600 mg/kg GOS; GOS + HCA = basal diets +600 mg/kg GOS + 200 mg/kg HCA, *n* = 6 for each group. * is significantly different (*p* < 0.05), whereas ns is not different (*p* > 0.05).

The relative abundance of bacteria at the phylum and genus level of sows at d 7 of lactation are presented in Figure 5. As shown, at the phylum level (Figure 5C), the abundance of *Planctomycetota* was higher (*p* < 0.05) in sows in the GOS + HCA group compared with sows in the CTRL group, but the abundance of *Cyanobacteria* was lower (*p* < 0.05) in sows in the GOS group compared with sows in the CTRL group. At the genus level (Figure 5D), compared with the CTRL group, the abundance of *g_Clostridia_UCG-014_unclassified* was increased (*p* < 0.05) by GOS+ HCA supplementation, whereas the abundance of *g_Clostridiales_unclassified* was increased (*p* < 0.05) by GOS alone supplementation.

### 3.5 Correlation between gut microbiota at genera level and the sows’ reproductive performance, concentrations of serum and colostrum metabolites and serum antioxidant index

**Figure 6:**
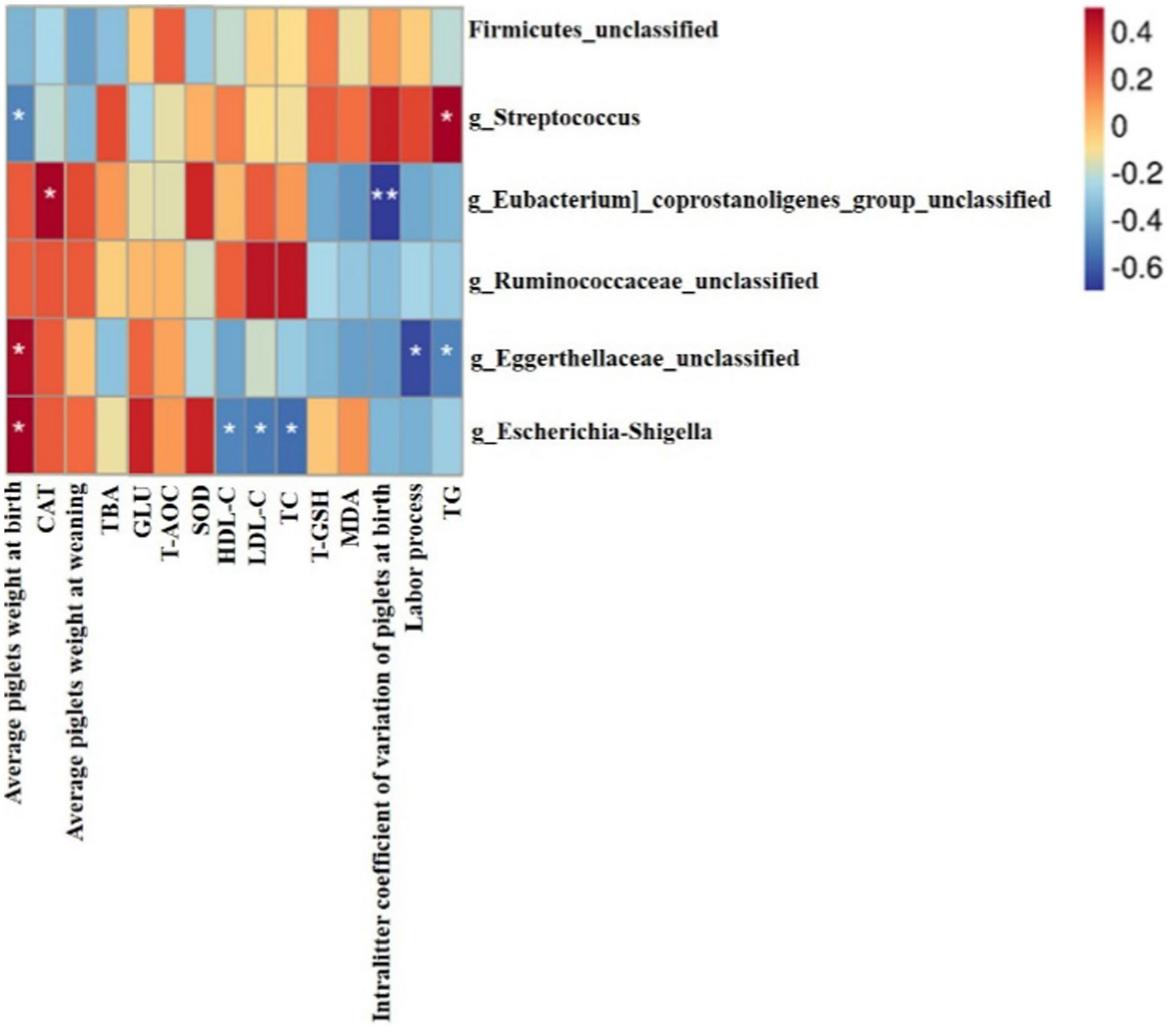
Heatmap of the spearman correlations between the gut microbiota significantly modified by different diets treatment and the detection indexes of sows at farrowing, *n* = 6 for each group. * *p* < 0.05; **p < 0.01.

As shown in Figure 6, the Spearman correlation matrix illustrated that the relative abundance of *g_Streptococcus* was positively correlated with serum TG of sows (*p* < 0.05), but negatively correlated with the average piglets weight at birth (*p* < 0.05). In contrast, *g_Eggerthellaceae_unclassified* and *g_Escherichia-Shigella* were positively correlated with the average piglet birth weight (*p* < 0.05). In addition, *g_Eggerthellaceae_unclassified* was negatively correlated with serum TG and the labor process of sows (*p* < 0.05). *g_Escherichia-Shigella* was negatively correlated with serum HDL-C, LDL-C and TC of sows (*p* < 0.05). *g_Eubacterium_coprostanoligenes_group_unclassified* was positively correlated with serum CAT of sows (*p* < 0.05), but was negatively correlated with intralitter coefficient of variation of piglets at birth (*p* < 0.01).

## 4 Discussion

Litter size and birth weight are pivotal traits in porcine production. This study examined the impact of dietary supplementation with galacto-oligosaccharides (GOS), both alone and in combination with hyocholic acids (HCA), on these traits in sows. Results indicated that such supplementation did not significantly alter the total number of piglets born. Prior research suggests that litter size is influenced primarily by fertilization rates and prenatal mortality in early gestation (Edwards et al., 2012). Moreover, the variation in birth weight within a litter is a key factor influencing piglet survival during lactation (Škorput et al., 2018). In this study, supplementation with GOS and GOS + Low/High HCA was observed to increase the average birth weight of piglets. This is comparable to earlier reports. Previous work has shown that supplementation with GOS for sows significantly decreased the number of stillborn piglets and increased the body weight and average daily weight gain of the offspring during the neonatal period (Wu et al., 2021). However, in this study, the impact of GOS and HCA supplementation on litter weight at weaning was minimal, potentially due to the specific dosages and environmental conditions affecting the sows (Quiniou and Noblet, 1999). Shorter farrowing durations, associated with improved piglet survival, were noted (Langendijk and Plush, 2019). Interestingly, while increased birth weight can typically extend the farrowing process due to increased mechanical resistance in the birth canal, the combination of GOS and high-dose HCA both increased average birth weight and shortened the farrowing duration. This could be attributed to the potential of GOS and HCA to modulate gut microbial communities, thereby alleviating compression from fecal matter in the birth canal and facilitating quicker delivery (Feyera et al., 2017; Lai et al., 2023). GOS has been reported to enhance the prevalence of beneficial gut bacteria, particularly *Bifidobacteria*, over pathogenic strains (Vulevi et al., 2008). Similarly, HCA has been shown to inhibit *Helicobacter pylori* growth (Itoh et al., 1999), suggesting that these supplements may alleviate constipation by restoring gut microbiota balance.

LDL-C, HDL-C, TG and TC are useful biomarkers for monitoring the absorption and transport of lipids, and the serum level of GLU can reflect energy and glucose metabolism (Shang et al., 2021). However, fat metabolism in serum could be regulated by oligosaccharide intake. It was found that supplementation of xylo-oligosaccharide reduced the serum concentration of triglyceride in nursery piglets (Hou et al., 2020). Similarly, it was found that supplementary feeding of GOS or combined with GOS and Low/High HCA in the perinatal period of sows could also significantly reduce serum TG. Bile acids have a beneficial effect in maintaining TG homeostasis. Research has indicated that bile acids, being the natural ligand of farnesoid X receptor (FXR), has the ability to activate FXR, modify the expression of genes related to triglyceride metabolism, and lower the level of triglycerides in plasma (Watanabe et al., 2004). This study also discovered that supplementing sows’ diets with GOS or a combination of GOS and Low/High HCA caused serum TG levels to drop and GLU levels to slightly rise. It is speculated that in order to fulfill the energy requirements of the sow’s delivery, this causes TG to be converted into GLU. Therefore, our experiment showed that the addition of the combination with GOS and Low/High HCA in sows’ diets could improve the efficiency of fat utilization, supplying more energy for the farrowing process. This is one of the explanations for the control group’s higher labor process than that of the GOS and HCA combined.

As the only energy and immune source for suckling piglets, the composition of colostrum and milk is critical for improving growth performance, especially immunoglobulin (Li et al., 2023). A previous study has shown that isomaltooligosaccharide could significantly improve the concentrations of colostrum IgA, IgG and IgM (Zhang et al., 2021). Paradoxically, another study reported that the sows fed mannan oligosaccharides did not affect IgA, IgG and IgM contents in colostrum (Duan et al., 2016). In our study, we found that only GOS supplementation did not affect the concentration of IgA in colostrum. The aforementioned circumstance might be linked to the quantity of oligosaccharides that were added, as well as to the structural units of oligosaccharides and the various ways in which they are connected. However, this study has demonstrated that adding Low/High HCA on the basics of the GOS supplement great boosted the amount of IgA in milk. This might have to do with the fact that bile acids have the ability to stimulate the growth of intestinal epithelial cells, which improves the absorption of amino acids and other nutrients while also increasing the amounts of raw materials available for the production of milk components, particularly milk proteins (Dossa et al., 2015). At the same times, it partly explains why average litter gain weight of the GOS + Low/High HCA groups was slightly heavier than that of the CTRL group.

The pregnant animals appear particularly vulnerable to reactive oxygen species because of the placenta’s extensive cell division and high metabolic activity (Sultana et al., 2023). Wang et al. (2022) has reported that the combination of GOS and *L. plantarum* supplementation in mice’s diets inhibited d-gal-induced oxidative stress. A recent study showed that early-life GOS supplements in suckling piglets’ diets alleviated the LPS-induced reactive oxygen species secretion, and inhibited the LPS-induced decrease of GSH-Px, T-AOC and SOD activities in serum (Tian et al., 2022). Consistent with these studies, the results of our experiment showed that activities of T-AOC and CAT in serum were significantly increased after GOS or GOS + High HCA treatment at farrowing. For the development of advantageous bacteria like *Lactobacilli* and *Bifidobacteria*, GOS is a high-quality nutritional source and an influential growth factor, which modulates the composition and amount of intestinal microbiota, restores the integrity of the intestinal barrier, and reduces oxygen free radicals (Schwab and Ganzle, 2011). Furthermore, GOS may be digested in the large intestine by bifidobacteria to create short-chain fatty acids, which lower intestinal pH values and prevent pathogenic bacteria from colonizing and growing (Fanaro et al., 2009). Simultaneously, the process by which exogenous bile acids can boost antioxidant activity in the body might be associated with the activation of nuclear factor erythroid-2-related factor 2 (Nrf2), which boosts the activity of enzymes like glutathione peroxidase, superoxide dismutase, and catalase in cells, and consequently lowers the levels of reactive oxygen species and malondialdehyde in cells (Li et al., 2020). Considering this observation, our research provides evidence of improvements in immunity and antioxidant ability in response to GOS or GOS + HCA supplementation for sows during the perinatal period.

The mammalian gastrointestinal tract is inhabited by trillions of microbes, which play a vital role in physiology, metabolism, immunity and so on. However, the composition of gut microbiota is implicated in various factors, including gut environment, nutrition and development stage. It is a remarkable fact that maternal gut microbiota can be passed to the developing fetus or newborn via the placenta, breast milk, or other routes (Koren et al., 2012). Nevertheless, the maternal will undergo metabolic syndrome during normal pregnancy, especially in late gestation, which decreases dramatically gut microbiota diversity and richness (Collado et al., 2010; Cheng et al., 2018). Recent studies showed that GOS had the promising potential to alleviate metabolic diseases by regulating the intestinal flora (Silk et al., 2009; Roselli et al., 2022). The dominating prebiotic functions of GOS are to selectively stimulate probiotic proliferation (*Bifidobacteria*, *Bacteroides*) and to produce short-chain fatty acids by fermenting (Li et al., 2015; Vulevic et al., 2015; Grimaldi et al., 2016). Similarly, bile acids, the compounds prevailingly regulating glucose and lipid metabolism, possess also potent antibacterial functions and play a pivotal role in shaping the microbial ecology of the gut by promoting the growth of bile acid-metabolizing bacteria and controlling the growth of bile acid-sensitive bacteria (Tian et al., 2020). Based on the above studies, we investigated the effects of GOS and combined GOS and HCA on the intestinal microbiome of sows during the perinatal period. Our results showed a profound alteration of the gut microbiota to be associated with different treatments and periods. At d 107 of gestation and at d 7 of lactation, sows fed GOS or combined GOS and HCA showed significantly higher gut microbial richness and *α*-diversity values than without them. Interestingly, at farrowing, the difference in the gut microbial richness and *α*-diversity values of sows among all groups was not found. The reason for this result may be an increased degree of metabolic disorder in sows at delivery, counteracting the positive effects of our current dose of added GOS and HCA (Cheng et al., 2018).

In the context of porcine pregnancy and lactation, *Firmicutes* and *Bacteroidetes* constitute the predominant bacterial phyla, a finding corroborated by our experimental data (Wang et al., 2021). Our research further revealed that dietary inclusion of galacto-oligosaccharides (GOS) and hyocholic acids (HCA) in sow diets resulted in a notable shift in gut microbiota composition. Specifically, there was a reduction in the relative abundance of the *Firmicutes* phylum and a corresponding increase in the *Bacteroidetes* phylum at farrowing, thereby lowering the *Firmicutes* to *Bacteroidetes* ratio, which is beneficial for improving intestinal microbiota disorders (Jiang et al., 2021). Meanwhile, *Bacteroides*, a dominant gut bacterium, plays a critical role in degrading polysaccharides and starches. This process not only supplies energy to the host but also maintains intestinal ecological balance through the fermentation of polysaccharides, leading to the production of short-chain fatty acids (Wang et al., 2017; Dejean et al., 2020). In our study, enrichment of *Ruminococcus* in sows after GOS and HCA supplementation might contribute to enhance nutritional metabolism, immune statues, and health outcomes (Blanton et al., 2016). These findings may elucidate the observed reduction in the duration of the labor process in sows fed with GOS or GOS + HCA supplemented diets. However, unexpectedly, the relative abundance of the *g_Escherichia_Shigella* increased significantly at farrowing when GOS + HCA was added to sows’ diet. As far as we are aware, intestinal inflammation can be brought on by a rise in the quantity of the *g_Escherichia_*Shigella (Sousa et al., 2010). During transit to the large intestine, bile acids undergo modifications to the steroid nucleus by some members of the gut microbiota, yielding deoxycholic acid (DCA) that the most typical secondary bile acids (Islam et al., 2011). Cao et al. (2017) has investigated that DCA-induced dysbiosis can increase the relative abundance of *g*_*Escheerichia-Shigella*, and disrupt intestinal barrier function. Our speculation is that this could be associated with the quantity of bile acids that have been introduced.

The intestinal microbiota plays a leading part in regulating mammalian lipid absorption, metabolism and storage (Wang et al., 2023). In our study, the relative abundance of *Streptococcus* was positively correlated with the content of serum TG. Meanwhile, the relative abundance of *Streptococcus* and the content of serum TG were significantly reduced, when sows were fed GOS diets. To some degree, it can be concluded from our research that GOS can ameliorate lipid metabolism disorder by modulating the composition of the gut microbiota.

## 5 Conclusion

In summary, this investigation demonstrated that the administration of galacto-oligosaccharides (GOS) in conjunction with hyocholic acids (HCA), to sows with nutrient restrictions during late gestation and lactation, further improved their antioxidant capacity and milk quality. However, further studies are warranted to elucidate the dose-dependent effects and causal relationships between the supplementation of GOS and HCA, the modulation of gut microbiota, and the reproductive performance of sows, including the underlying biological mechanisms.

## Data availability statement

The data presented in the study are deposited in the NCBI repository, accession number PRJNA1117460.

## Ethics statement

The animal studies were approved by animal care and use committee certificate of approval this is to certify that project no: 20230423 project title: study on the mediating mechanism of gut microbiota in the regulation of liver lipid metabolism in periparturient sows by dietary fiber. Principal researchers: ZF and BT the project proposal submitted on 1-January-2021 was reviewed by three members of the animal care and use committee, and was approved on 9-March-2021. It is the Principal researcher’s responsibility to ensure that all researchers associated with this project are aware of the conditions of approval and which documents have been approved. The principal researcher is required to notify the secretary of the animal care and use committee, via amendment or progress report, of any significant change to the project and the reason for that change, including an indication of ethical implications (if any); any other unforeseen events or unexpected developments that merit notification; the inability of the principal researcher to continue in that role, or any other change in research personnel involved in the project; Signed by: Dr. He Xi. the studies were conducted in accordance with the local legislation and institutional requirements. Written informed consent was obtained from the owners for the participation of their animals in this study.

## Author contributions

JYu: Conceptualization, Data curation, Investigation, Methodology, Software, Writing – original draft, Writing – review & editing. JW: Conceptualization, Data curation, Investigation, Methodology, Software, Writing – original draft, Writing – review & editing. CC: Investigation, Supervision, Writing – review & editing. JG: Supervision, Writing – review & editing. JC: Investigation, Supervision, Writing – review & editing. JYi: Conceptualization, Supervision, Writing – review & editing. SW: Conceptualization, Supervision, Writing – review & editing. PH: Conceptualization, Supervision, Writing – review & editing. BT: Conceptualization, Methodology, Supervision, Writing – review & editing. ZF: Conceptualization, Data curation, Methodology, Supervision, Writing – review & editing.
