# Supplementary material for: Maternal intervention with a combination of galacto-oligosaccharides and hyocholic acids during late gestation and lactation increased the reproductive performance, colostrum composition, antioxidant and altered intestinal microflora in sows
Source: Front Microbiol. 2024 Jun 12;15:1367877. doi: 10.3389/fmicb.2024.1367877 (PMC11199897; doi:10.3389/fmicb.2024.1367877)
Supplement: Supplementary file 1 [file Data_Sheet_1.docx]

Supplementary Material

# Supplementary Data

Table S1 Raw reads and selected valid sequences in each sample

| Sample name^1^ | At d 107 of gestation | | | At farrowing | | | At d 7 of lactation | | |
| --- | --- | --- | --- | --- | --- | --- | --- | --- | --- |
|  | Raw_Tags | Valid_Tags | OUTs | Raw_Tags | Valid_Tags | OUTs | Raw_Tags | Valid_Tags | OUTs |
| CTRL-1 | 85,340 | 75,682 | 556 | 84,958 | 75,179 | 910 | 85,889 | 76,855 | 618 |
| CTRL-2 | 85,340 | 75,571 | 746 | 81,904 | 70,443 | 513 | 87,082 | 78,468 | 815 |
| CTRL-3 | 86,944 | 76,611 | 632 | 81,209 | 70,769 | 630 | 82,684 | 74,724 | 840 |
| CTRL-4 | 86,609 | 72,766 | 398 | 81,881 | 73,075 | 984 | 80,814 | 72,874 | 830 |
| CTRL-5 | 85,911 | 75,763 | 586 | 85,541 | 75,859 | 999 | 81,676 | 74,316 | 828 |
| CTRL-6 | 87,903 | 77,741 | 828 | 82,869 | 72,485 | 832 | 80,496 | 72,834 | 1,014 |
| GOS-1 | 86,701 | 75,128 | 739 | 83,495 | 74,841 | 923 | 80,488 | 70,926 | 582 |
| GOS-2 | 83,926 | 72,035 | 690 | 84,700 | 75,819 | 938 | 82,001 | 72,192 | 962 |
| COS-3 | 82,344 | 73,388 | 871 | 86,661 | 76,190 | 725 | 85,487 | 75,367 | 886 |
| COS-4 | 84,298 | 74,857 | 795 | 82,375 | 72,890 | 1,044 | 85,772 | 74,378 | 523 |
| COS-5 | 82,775 | 74,185 | 816 | 85,450 | 75,870 | 929 | 85,274 | 75,928 | 761 |
| COS-6 | 84,841 | 73,964 | 672 | 84,534 | 74,842 | 689 | 83,633 | 74,568 | 915 |
| GOS+HCA-1 | 82,297 | 72,604 | 900 | 80,968 | 70,913 | 692 | 85,142 | 76,278 | 1,013 |
| GOS+HCA-2 | 87,970 | 76,326 | 456 | 83,367 | 74,409 | 1,004 | 86,704 | 76,881 | 990 |
| GOS+HCA-3 | 86,225 | 76,544 | 934 | 85,015 | 75,702 | 883 | 82,089 | 74,117 | 794 |
| GOS+HCA-4 | 86,481 | 76,725 | 977 | 84,614 | 73,911 | 900 | 85,447 | 76,926 | 1,039 |
| GOS+HCA-5 | 85,257 | 759,971 | 936 | 86,135 | 77,153 | 963 | 85,889 | 76,969 | 939 |
| GOS+HCA-6 | 83,680 | 74,798 | 922 | 84,764 | 75,719 | 1,072 | 83,723 | 72,665 | 464 |

^1^ CTRL= basal diets; GOS= basal diets + 600 mg/kg GOS; GOS+HCA=basal diets + 600 mg/kg + 200 mg/kg HCA.
